# Low-Temperature Calcium Phosphate Ceramics Can Modulate Monocytes and Macrophages Inflammatory Response In Vitro

**DOI:** 10.3390/biomedicines12020263

**Published:** 2024-01-24

**Authors:** Vladislav V. Minaychev, Polina V. Smirnova, Margarita I. Kobyakova, Anastasia Yu. Teterina, Igor V. Smirnov, Vladimir D. Skirda, Artem S. Alexandrov, Marat R. Gafurov, Mikhail A. Shlykov, Kira V. Pyatina, Anatoliy S. Senotov, Pavel S. Salynkin, Roman S. Fadeev, Vladimir S. Komlev, Irina S. Fadeeva

**Affiliations:** 1 Institute of Theoretical and Experimental Biophysics, Russian Academy of Sciences, 142290 Pushchino, Russia; vminaychev@gmail.com (V.V.M.); kobyakovami@gmail.com (M.I.K.); a.s.senotov@gmail.com (A.S.S.); aurin.fad@gmail.com (I.S.F.); 2 Baikov Institute of Metallurgy and Materials Science, Russian Academy of Sciences, Leninskiy Prospect 49, 119334 Moscow, Russia; smirnova-imet@mail.ru (P.V.S.); teterina_imet@mail.ru (A.Y.T.); ceshakov@gmail.com (M.A.S.); 3 Institute of Physics, Kazan Federal University, Kremlyovskaya St. 18, 420008 Kazan, Russia; kazanvs@mail.ru (V.D.S.); marat.gafurov@kpfu.ru (M.R.G.)

**Keywords:** biomaterials, calcium phosphates, biocompatibility, regenerative medicine, cell technology, tissue engineering, bone tissue engineering

## Abstract

Creating bioactive materials for bone tissue regeneration and augmentation remains a pertinent challenge. One of the most promising and rapidly advancing approaches involves the use of low-temperature ceramics that closely mimic the natural composition of the extracellular matrix of native bone tissue, such as Hydroxyapatite (HAp) and its phase precursors (Dicalcium Phosphate Dihydrate—DCPD, Octacalcium Phosphate—OCP, etc.). However, despite significant scientific interest, the current knowledge and understanding remain limited regarding the impact of these ceramics not only on reparative histogenesis processes but also on the immunostimulation and initiation of local aseptic inflammation leading to material rejection. Using the stable cell models of monocyte-like (THP-1ATRA) and macrophage-like (THP-1PMA) cells under the conditions of LPS-induced model inflammation in vitro, the influence of DCPD, OCP, and HAp on cell viability, ROS and intracellular NO production, phagocytosis, and the secretion of pro-inflammatory cytokines was assessed. The results demonstrate that all investigated ceramic particles exhibit biological activity toward human macrophage and monocyte cells in vitro, potentially providing conditions necessary for bone tissue restoration/regeneration in the peri-implant environment in vivo. Among the studied ceramics, DCPD appears to be the most preferable for implantation in patients with latent inflammation or unpredictable immune status, as this ceramic had the most favorable overall impact on the investigated cellular models.

## 1. Introduction

Despite the abundance of proposed bone substitutes, there is a lack of truly effective materials for bone tissue regeneration and, particularly, augmentation among them. The desired properties can only be provided by bone autografts, the availability of which is extremely limited by the patient’s own capabilities [1,2,3,4,5]. In turn, the proposed synthetic calcium phosphates (CPs) often not only fail to promote tissue regeneration but also hinder it, initiating rejection processes and the development of material-associated peri-focal bone resorption of patients. The absence of reliable and effective materials is particularly acute in the development of bone grafting surgery (vertical augmentation) and reconstructive surgery for critically sized bone defects, as no necessary solutions have been proposed to address these acute problems.

It is known that the physicochemical properties of calcium phosphate substitute materials play a central role in their behavior in the body. By varying these properties, materials of different types and degrees of biocompatibility can be obtained. From one perspective, controlled variation in the material properties of CPs allows the production of materials of various types, shapes, and purposes, including through combination with natural biopolymers [6,7] or their use as effective carriers of therapeutic agents [8]. However, such dependence on physicochemical properties ensures a wide variability of biological effects, from successful integration into the body to rejection, causing harm that exceeds the initial problem of the patient [9,10,11,12].

The existing problem of the variability in the biological effects of CPs raises the question of the need to develop new approaches to the creation of CP materials and the study of their biological properties. The recent paradigm shift in modern biomedical materials science indicates clear trends in transitioning from high-temperature ceramics to biomimetic nature-like materials (synthesized at physiological temperatures, ≤40 °C), i.e., creating CPs with a structure and composition as close as possible to the native mineral of bone tissue, inducing homing, migration, and differentiation of precursor cells at the implantation site, thus enabling effective directed tissue regeneration and augmentation [13,14,15,16,17,18]. Increasing attention is paid to the study of various physiological precursors of hydroxyapatite (HAp), such as dicalcium phosphate dihydrate (DCPD) and octacalcium phosphate (OCP). Interest in such CPs is justified by several studies demonstrating their greater biological activity compared to crystalline hydroxyapatite due to greater solubility and the ability to undergo phase transformation in the body into native bone tissue hydroxyapatite. In our opinion, the use of CPs synthesized under physiological conditions can significantly increase the biocompatibility of CP biomaterials [19,20,21].

Another important aspect of CP osteointegration is their interaction with immune cells, primarily with immune cell macrophages. Thus, one of the central problems limiting the use of various CP materials is the development of uncontrolled inflammatory reactions. Consequently, the study of the interaction of CPs with immune cells is of increasing interest. It is known that macrophages and their monocyte precursors play an important role in all stages of bone tissue remodeling and regeneration, as well as determine the body’s response to implanted material by forming an appropriate proinflammatory and/or pro-regenerative microenvironment [22,23,24]. The influence of hydroxyapatite precursors on the behavior of these cells remains largely unstudied, which is partly due to the complexity of such studies and the difficulty in choosing experiment models adequately reflecting the ongoing biological processes. The lack of direct access to a broad base of healthy donors for ordinary researchers, the complexity of isolation, and the variability of the biological response of primary cell cultures to materials, associated with the spatiotemporal sensitivity of the donor’s body, make it difficult to use them to assess the proinflammatory and pro-regenerative potential of materials in vitro and also complicates the interpretation of the obtained data.

In this regard, the use of immortalized cell cultures appears to be the most adequate for studying the general principles of material interaction with immune cells. THP-1 represents a line of human leukemia monocyte cells that are widely used to study the functions of monocytes/macrophages [25,26,27,28]. This cell line has become a widely accepted model for evaluating the modulation of monocyte and macrophage activity due to its similarity to primary monocytes and macrophages in terms of differentiation, reproducibility, low variability of cell phenotypes due to homogeneous genetic background, and a long period of use without the loss of sensitivity or activity [28,29,30,31]. All these properties make THP-1 cells more preferable compared to cell lines PBMC and other primary cells—monocytes and macrophages [28,31].

The aim of this study was to investigate the influence of physiological participants in the bone mineralization process—DCPD, OCP, and HAp, obtained using a low-temperature chemical transformation approach—on the proinflammatory properties of monocyte-like and macrophage-like cells in vitro.

## 2. Materials and Methods

### 2.1. Characterization and Synthesis Procedure

#### 2.1.1. Materials Synthesis

CP powders, such as DCPD (dicalcium phosphate dihydrate), OCP (octacalcium phosphate), and HAp (hydroxyapatite), were prepared using low-temperature approaches. α-tricalcium phosphate (α-TCP) was used as the initial material. α-TCP powder was soaked in a buffer solution composed of 0.15 M L-glutamic acid and 1.5 M sodium acetate (pH = 5.5 ± 0.2, sample weight/solution weight ratio = 1/100) for 24 h at 35 ± 2 °C with constant stirring; DCPD underwent hydrolysis. 

OCP was prepared from DCPD powder in a 1.5 M sodium acetate solution (pH = 9.0 ± 0.2, sample weight/solution weight ratio = 1/100) for 24 h at 35 ± 2 °C with constant stirring.

The hydrolysis of OCP samples into hydroxyapatite (HAp) was performed in 2 M sodium acetate solution (pH = 10.0 ± 0.2, sample weight/liquid weight ratio = 1/100) for 24 h at 40 ± 2 °C with constant stirring. 

#### 2.1.2. Phase Composition

The phase composition of the dried (37 °C for 24 h) powders was studied using a Shimadzu XRD-6000 diffractometer. Full profile analysis was performed using Jana 2006 software and JCPDS 2003 data bank to analyze the diffraction patterns and define the crystalline structures. 

Nicolet Avatar 330 FT-IR spectrometer was used to determine the transmission spectra of dried CP samples in the range of 4000–400 cm^−1^ with a resolution of 2 cm^−1^. The mixture with potassium bromide was used.

#### 2.1.3. Microstructure and Morphology

SEM (Tescan VEGA III, Brno, Czech Republic) was used to examine the microstructures and morphologies of the samples. Enhanced scanning electron microscopy imaging was performed by gold coating the samples with Q150R Quorum Technologies (Lewes, UK). The material surfaces were imaged at pressures of 7.3 × 10^−2^ Pa in the column and 1.5 × 10^−1^ Pa in the chamber. 

#### 2.1.4. Nuclear Magnetic Resonance 

^1^H and ^31^P MAS NMR spectra were obtained using Bruker AVANCE III 400 spectrometer (Karlsruhe, Germany) at resonant frequencies of 400.27 MHz and 162.06 MHz, respectively, in the magnetic field of about 9.5 T. Spectra were obtained using free induction decay (FID), FID with proton decoupling and Carr–Purcell–Meiboom–Gill (CPMG) pulse sequences. The duration of the π/2 RF-pulse was 2.5 and 3.4 μs for ^1^H and ^31^P channels, respectively; the repetition time was 5 s for both nuclei. Powder samples were densely packed in a 4 mm zirconium oxide rotor and spun up to a rotation frequency of 7 kHz for the solid-state magic angle spinning (MAS) NMR. All NMR measurements were performed at a room temperature of 293 K.

### 2.2. In Vitro Studies

#### 2.2.1. Cell Culture

The study utilized monoblast-like cells from the THP-1 cell line obtained from ATCC (Wesel, Germany). Cells were cultured at 37 °C in a humidified atmosphere of 5% CO_2_ in RPMI-1640 medium containing 10% fetal bovine serum, 2-mercaptoethanol (0.05 mM), and gentamicin sulfate (40 μg/mL). Mycoplasma infection has been detected in cell cultures using a MycoFluor^TM^ Detection Kit (Thermo Scientific, Waltham, MA, USA), and no mycoplasma infection was found.

Monocyte-like THP-1ATRA cells were derived by treating THP-1 cells with ATRA (all-trans retinoic acid) (Merck, Hamburg, Germany). To do this, THP-1 cells were treated with 1 μM ATRA for 72 h [32,33].

THP-1PMA macrophage-like cells were derived by treating THP-1 cells with phorbol 12-myristate 13-acetate (PMA). To do this, THP-1 cells were treated with 100 nM PMA for 72 h and then washed three times with culture medium [34]. The cells were detached from the surface of the culture plastic using a 0.05% EDTA-trypsin solution (PanEko, Moscow, Russia).

Human monocytes (Cell Applications Inc., San Diego, CA, USA) were used to generate peripheral blood-derived macrophages (PBDMs). Monocytes were grown in Dulbecco’s modified Eagle’s medium (DMEM) supplemented with gentamicin sulfate (40 μg/mL) and 10% fetal bovine serum (FBS) at 37 °C in a moist atmosphere of 5% CO_2_ for three days from the time of plating. On day 4, the culture medium was replaced with fresh DMEM containing 2% FBS, and the cells were cultured for another 7 days. Using a 0.05% EDTA-trypsin solution (PanEko, Moscow, Russia), the cells were separated from the surface of the culture plastic.

The co-culture continued for an additional 72 h. DCPD, OCP, and HAp powders were pre-sterilized with an ethanol solution at a concentration of 75% using the specified technique [19].

#### 2.2.2. Cell Immunoprofiling

To investigate the expression of CD cell monocyte and macrophage markers, cells were stained with the following monoclonal antibodies: APC anti-human CD11b; FITC anti-human CD11c; FITC anti-human CD14; PE anti-human CD36; PE anti-human CD64; FITC anti-human CD68; PE anti-human CD284; PE anti-human HLA-DR. Nonspecific binding was determined by staining cells with isotype control antibodies: APC Mouse IgG1 k isotype Ctrl; FITC Mouse IgG1 k isotype Ctrl; PE Mouse IgG1 k isotype; and PE Mouse IgG2a k isotype. Cells were stained for 30 min in the dark and then fixed with 2% paraformaldehyde. The analysis was performed using a BD Accuri C6 flow cytometer (BD/Fisher Sci., Franklin Lakes, NJ, USA) [32,34].

#### 2.2.3. Cell Viability Testing

AlamarBlue (Invitrogen, Carlsbad, CA, USA) was used to measure the cell viability after 72 h of incubation with DCPD, OCP, and HAp. The cells were incubated with 100 μg/mL AlamarBlue for 4 h at 37 °C in a moist environment of 5% CO_2_, and the mean fluorescence intensity (MFI) of the resultant resofurin product was measured using an Infinite^®^200 microplate reader (Tecan Group Ltd., Männedorf, Switzerland): Ex530 nm/Em 595 nm. The vitality of the control cells that had not been treated with DCPD, OCP, or HAp was assumed to be 100%. Cell viability was measured as a percentage relative to the control using the following formula: Cell viability (%) = (MFI cells after incubation with DCPD, OCP, and HAp/MFI of control cells) × 100% [19,33].

#### 2.2.4. Determination of Cytoplasmic Granularity

The granularity of the cytoplasm of the cells was determined by lateral light scattering (SSC) on a BD Accuri C6 Cell Analyzer (BD/Fisher Sci., Franklin Lakes, NJ, USA).

#### 2.2.5. Cytochemical Staining by Romanovsky-Giemsa

Cells were stained sequentially in Diachym-Hemistein-R solution (Abris Plus, Krasnodar, Russia) for 20 min, and in Giemsa solution (Sigma-Aldrich, Hamburg, Germany) for 10 min. The samples were then washed under running water for 1 min. Micrographs of the stained preparations were obtained using a microscope fluorescent station Eclipse Ti-E (Nikon, Tokyo, Japan).

#### 2.2.6. Analysis of Mitochondrial Mass

To determine the mitochondrial mass, cells were stained with 100 nM MitoTracker Green FM (Fisher Sci., Waltham, MA, USA). In a CO_2_ incubator (Binder GmbH, Tuttlingen, Germany), the cells were incubated for 30 min. Determination was carried out using a BD Accuri C6 Cell Analyzer (BD/Fisher Scientific, Franklin Lakes, NJ, USA).

#### 2.2.7. LysoTracker Staining

After 72 h of co-incubation with DCPD, OCP, and HAp, the cells were stained for 30 min in a CO_2_ incubator with 50 nM LysoTracker Green DND-26 (Fisher Sci., Waltham, MA, USA). For 4 h, control cells were preincubated with 50 M chloroquine (MerckMillipore, St. Louis, MO, USA). Cell fluorescence was analyzed using a BD Accuri C6 Cell Analyzer (BD/Fisher Sci., Franklin Lakes, NJ, USA) [33].

#### 2.2.8. ROS Production Assay

To determine constitutive and LPS-induced intracellular reactive oxygen species production, after 72 h of co-incubation with DCPD, OCP, and Hap, cells were stained with DCFH-DA (Sigma-Aldrich, St. Louis, MO, USA). To determine the constitutive intracellular production of reactive oxygen species, cells were stained with 40 μM DCFH-DA for 30 min under CO_2_ incubator conditions. To study inducible intracellular production of reactive oxygen species, cells were preincubated with 10 μg/mL LPS from *E. coli* O111:B4 (MerckMillipore, Chemicon^®^, Rolling Meadows, IL, USA) for 24 h. Control cells were preincubated with 1 mM hydrogen peroxide (HP) (Sigma-Aldrich, St. Louis, MO, USA) for 30 min. Cell fluorescence analysis was performed using a BD Accuri C6 Cell Analyzer (BD/Fisher Sci., Franklin Lakes, NJ, USA) [19,33].

#### 2.2.9. Intracellular NO Assay

To assess constitutive and LPS-induced intracellular nitric oxide (NO) production, after 72 h of co-incubation with DCPD, OCP, and Hap, cells were stained with DAF-FM DA (Fisher Sci., Waltham, MA, USA). Cells were stained with 5 M DAF-FM DA for 40 min before being washed with fresh growth medium and cultured in a CO_2_ incubator for another 30 min. Cells were pre-incubated for 24 h with 10 μg/mL LPS from *E. coli* O111:B4 to explore inducible NO generation. Cell fluorescence was analyzed using a BD Accuri C6 Cell Analyzer (BD/Fisher Sci., Franklin Lakes, NJ, USA) [33].

#### 2.2.10. Phagocytosis Assay

To assess phagocytic activity, the number of phagocytic cells and the mean fluorescence intensity (MIF) of pHrodo Green *E. coli* (Fisher Sci., Waltham, MA, USA) in phagocytic cells were determined. After 72 h of co-incubation with DCPD, OCP, and HAp, cells were incubated with 1 mg/mL pHrodo Green *E. coli* (Fisher Sci., Waltham, MA, USA) for 2 h after cell incubation in a CO_2_ incubator. To control for nonspecific staining, cells were preincubated with 10 μg/mL cytochalasin D (MerckMillipore, St. Louis, MO, USA). Measurements were performed using a BD Accuri C6 Cell Analyzer (BD/Fisher Sci., Franklin Lakes, NJ, USA) [33].

#### 2.2.11. Analysis of TNF-α, IL-1β, and IL-6 Cytokine Secretion

To assess the inducible secretion of proinflammatory cytokines (TNF-α, IL-1β, and IL-6), cells were pre-incubated with 10 μg/mL lipopolysaccharide (LPS) for 24 h. For the determination of cytokine secretion after 72 h of cell cultivation with DCPD, OCP, and HAp powder samples, cells were harvested from the culture dish, centrifuged (300× *g*, 5 min), and the supernatant was used for analysis. The secretion of TNF-α, IL-1β, and IL-6 was assessed using commercial kits: Interleukin-1beta-IFA-BEST, Interleukin-6-IFA-BEST, and Alpha-IFN-IFA-BEST (Vector-Best, Moscow, Russia), following the manufacturer’s instructions. Optical density was measured at a wavelength of 450 nm using an iMark microplate reader (Bio-Rad, Hercules, CA, USA).

#### 2.2.12. Statistical Analysis

The results of the conducted study are presented as the mean ± standard deviation. All experiments used statistical samples with no fewer than 4 observations (*n* ≥ 4). 

The collected findings were statistically analyzed using Python 3 programming language (ver. 3.10.6) in the Spyder framework for development (ver. 5.4.1), with Pandas (ver. 1.5.3), Numpy (ver. 1.23.5), and Scipy (ver. 1.10.0) tools. Multiple comparisons of the investigated groups were conducted using one-way ANOVA, followed by Tukey’s post hoc test for comparing experimental groups relative to each other. At *p* < 0.05, differences between groups were determined to be statistically significant. Multiple comparisons were conducted using the scikit–posthocs package (version 0.7.0) [35]. Before conducting multiple comparisons, tests for normality of distribution and equality of variances were performed using the Shapiro–Wilk and Brown–Forsythe tests, respectively, with the scipy package.

The graphical representation of the obtained results was created using the Python 3 programming language (ver. 3.10.6) with the Matplotlib (ver. 3.7.0) and Seaborn (ver. 0.12.2) packages.

## 3. Results

### 3.1. The Physico-Chemical Characteristics of Low-Temperature CPs

**Figure 1:**
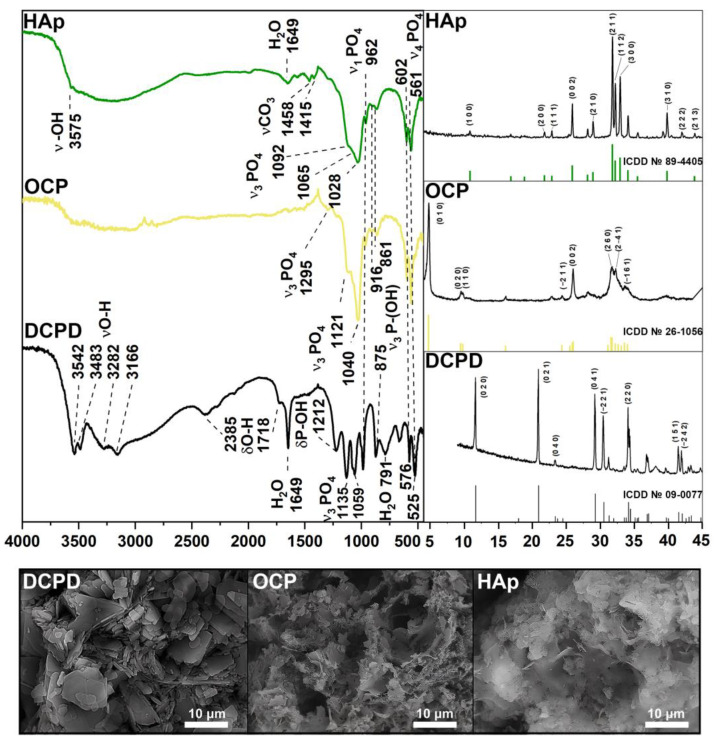
XRD, FTIR spectra, and SEM images of materials DCPD, OCP, and HAp.

According to the XRD and FTIR results (Figure 1), a single-phase crystalline DCPD was obtained. The IR-spectrum of DCPD shows bending modes of the PO_4_^2−^ group at 875 and 987 cm^−1^ (ν1); 1059 and 1135 cm^−1^ (ν3); 525, 576 and 662 cm^−1^ (ν4). Peaks assigned to the P-OH bound are noted at 875 and 1212 cm^−1^. The stretching modes of the O-H bond of lattice water appear at 3550–3160 cm^−1^, with bending modes at 1649 cm^−1^. The presence of absorbed water was observed at 2385 and 1718 cm^−1^. The XRD spectrum corresponds to card No. 9-0077 of the XRD base ICDD (Powder Diffraction File, Pennsylvania: JCPDS, 2011). The main XRD peaks of DCPD (020), (021), (041) and (220) are noted. The microstructure of the obtained samples is typical for DCPD: plate-like crystals with an average size of 2–20 µm.

FTIR and XRD spectra confirmed the high-crystallinity and single-phase nature of OCP. The presence of the intense bands at 1121 and 1040 cm^−1^ corresponding to the ν3 mode of HPO_4_^2−^ and PO_4_^3−^ [P−O stretching in phosphate (PO_4_^3−^) and hydrogen phosphate (HPO_4_^2−^)], and an HPO_4_^2−^ band at 916 cm^−1^ (P−O stretching in HPO_4_^2−^) is typical of an OCP structure. The 602 cm^−1^ and 561 cm^−1^ sharp P−O peaks (P−O deformation in PO_4_^3−^) belong to the ν4 mode of PO_4_^3−^. The bands at 862 cm^−1^ and 961 cm^−1^ belong to HPO_4_^2−^ stretch groups (P–OH). The primary vibration peaks of the PO_4_^3−^ and HPO_4_^2−^ groups are indicative of the OCP structure. the XRD results are in good agreement with the FTIR spectrum. The phase compositions of the samples with the major peaks (010), (020), (002), (260), and (2–41) conform to the XRD base ICDD card No 26-1056. The standard chaotic structure of OCP with thin plates can be observed on SEM images. 

Pure and high-crystalline HAp powder was obtained according to Figure 1. A sharp peak at 3575 cm^−1^ belongs to the OH^−^ ion, which is a characteristic of HAp. The same modes of PO_4_^3−^ groups remain unchanged (ν1 at 961 cm^−1^, ν3 at 1028, 1065 and 1092 cm^−1^, ν4 at 561 cm^−1^, 602 cm^−1^). The relative decrease of the HPO_4_^2−^ peaks at 861, 917 and 1212 cm^−1^ can be observed. The stretching band of absorbed water occurs at 1649 cm^−1^. Bands at 1415 and 1458 cm^−1^ are assigned to the stretching vibrations of the CO_3_^2−^ group. According to the XRD results, HAp peaks (100), (200), (002), (211), (112), (300) и (310) accurately correspond to card No. 89-4405. As seen, HAp crystals form thin needles and plates. All IR results are in good agreement with the literature data [36]. 

### 3.2. Solid-State MAS NMR

**Figure 2:**
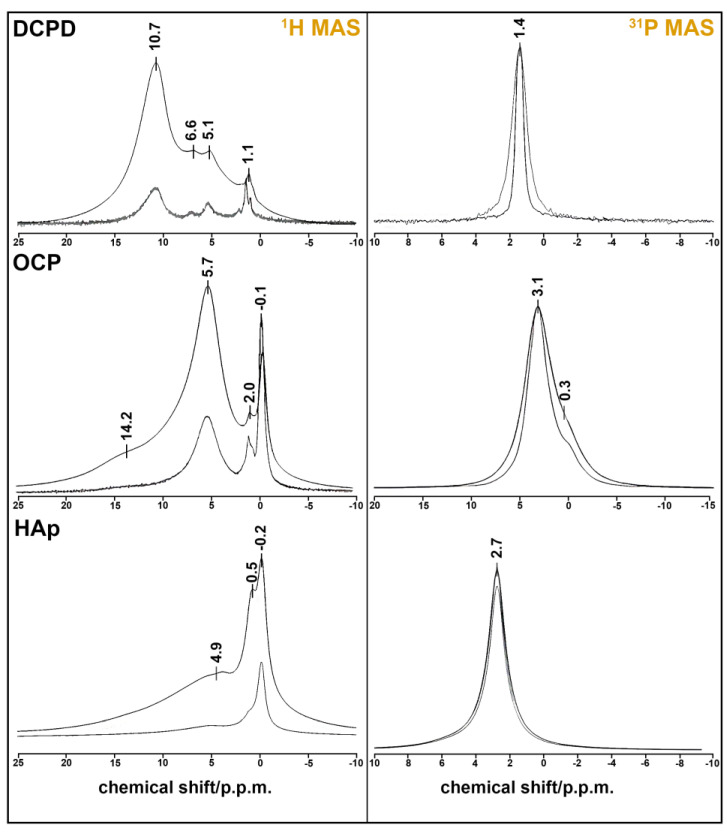
NMR spectra of CPS: ^1^H MAS spectra (**left**) obtained from FID (ZG) and spin echo (CPMG) at τ = 100 µs; ^31^P MAS spectra (**right**) obtained in the absence (ZG) and presence (HPDEC) of proton suppression.

The results of the solid-state HMR MAS experiment confirm our material descriptions (Figure 2). Single-phase samples were obtained in every case. DCPD has monoclinic syngony, so all HPO_4_^2−^ ions are crystallography equivalent, as seen at ^31^P MAS spectrum with one sharp peak at 1.4 ppm. The figure displays a comparison of the ^31^P NMR spectra of the DCPD sample obtained in the absence (ZG) and presence (HPDEC) of proton suppression. The difference in the linewidth of these spectra indicates the presence of a dipole–dipole magnetic coupling ^1^H-^31^P.

The ^31^H MAS spectrum consists of three unresolved peaks at 5.1, 6.6, and 10.7 ppm. One of them belongs to HPO_4_^2−^ proton (10.7 ppm), and two of them are attributed to the four protons of two molecules of lattice water [37]. The sharp peak at 1.1 ppm may belong to absorbed water, which is also visible at 1649, 1718, and 2385 cm^−1^ on the IR spectrum of DCPD (Figure 2).

OCP crystal has the lowest symmetry (P1), and the unit cell has four inequivalent PO_4_^3−^ groups and two inequivalent HPO_4_^2−^ groups. They appear as two unresolved peaks at 0.3 (HPO_4_^2−^) and 3.1 (PO_4_^3−^) ppm at the ^31^P spectrum [38]. Two sharp peaks at −0.1 and 2.0 ppm on the ^1^H MAS spectrum belong to the five inequivalent water molecules of the OCP formula unit [39]. The broad line at 5.7 ppm can be ascribed to the adsorbed water by the powder sample. Protons from the two HPO_4_^3−^ groups form a very broad peak at ≈14.2 ppm.

HAp crystallizes in hexagonal syngony and has one sharp peak at 2.7 ppm on the ^31^P MAS spectrum. The proton spectrum of HAp also has one sharp peak at −0.2 ppm, which belongs to the OH^−^ group. Two broad peaks at 0.5 and 4.9 p.p.m. may be attributed to the absorbed water, whose mode can be seen at 1649 cm^−1^ in Figure 1. This assumption may be also reinforced by the fact that the phosphorus spectrum shows the absence of CP impurities (there is only one peak of six equivalent PO_4_^3−^ groups).

### 3.3. THP-1 ATRA and THP-1 PMA Cells Have Phenotypic Features That Are Characteristic of Human Monocytes and Macrophages

**Figure 3:**
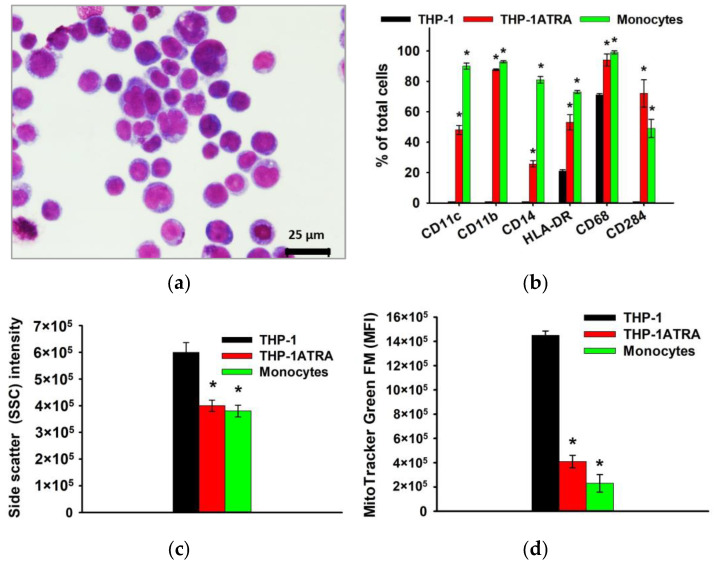
Phenotype of THP-1ATRA monocyte-like cells in comparison with THP-1 cells and human monocytes. Morphology of THP-1ATRA monocyte-like cells; Romanovsky–Giemsa staining, light microscopy (**a**). CD markers of monocyte differentiation in THP-1ATRA cells in comparison to THP-1 cells and human monocytes (**b**). Granularity of THP-1, THP-1ATRA cells, and human monocytes (**c**). The ordinate axis represents the average value of the intensity of lateral light scattering by the cell in relative units (SSC). The relative content of mitochondria in THP-1, THP-1ATRA cells, and human monocytes (**d**). The mean fluorescence intensity of cells (arb. units) infused with MitoTracker Green FM is referred to as the MFI. The data are shown as the mean ± SD (*n* ≥ 3). * *p* < 0.05 in comparison to THP-1 cells.

As already noted above, macrophages and their precursors, monocytes, play an important role at all stages of remodeling and regeneration of bone tissue, as well as determine the body’s response to the implanted material, due to the formation of appropriate pro-inflammatory and/or pro-regenerative microenvironment. In this regard, further in the work, the influence of physiological participants in the biomineralization process—DCPD, OCP, and HAp, obtained using the low-temperature chemical transformation approach—on the proinflammatory properties of macrophage-like and monocyte-like cells was studied. THP-1 cells were treated with phorbol 12-myristate 13-acetate (PMA) to produce macrophage-like THP-1PMA cells. Trans-retinoic acid treatment of THP-1 cells resulted in monocyte-like THP-1ATRA cells. In the first stage, we characterized the phenotype of THP-1ATRA and THP-1PMA cells. To characterize the phenotype of THP-1PMA and THP-1ATRA cells, we compared morphological features, immune phenotype, intracellular nitric oxide production, mitochondrial mass, and the granularity of THP-1ATRA and THP-1PMA and THP-1 cells, as well as PBDM monocytes and macrophages. THP-1ATRA cells had phenotypic features characteristic of human monocytes. Thus, THP-1ATRA increased the number of cells carrying CD11b, CD11c, CD14, CD68, CD284, and HLA-DR receptors, as well as decreased mitochondrial mass and granularity in comparison with THP-1 cells, which was also characteristic of peripheral human monocytes (Figure 3).

**Figure 4:**
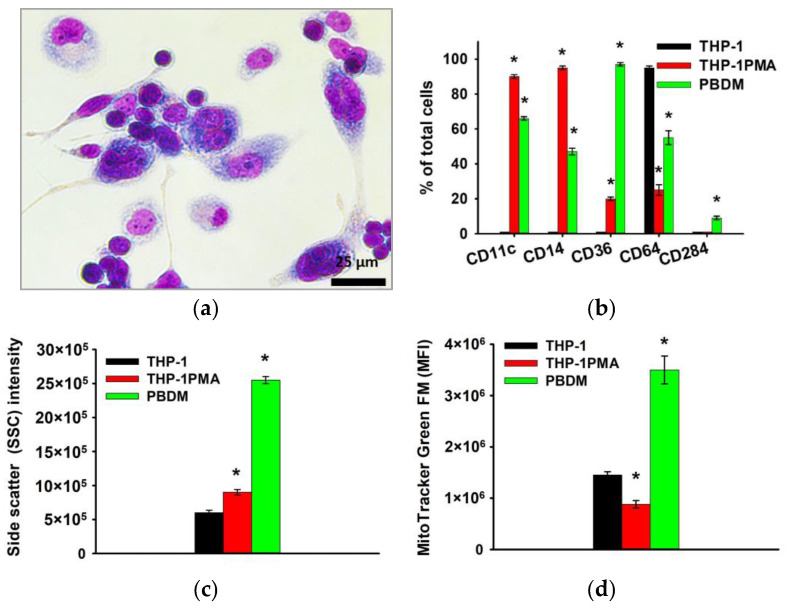
Phenotype of THP-1PMA monocyte-like cells in comparison with THP-1 cells and PBDM macrophages. Morphology of THP-1PMA macrophage-like cells; Romanovsky–Giemsa staining, light microscopy (**a**). CD markers of monocyte differentiation in THP-1PMA cells in comparison to THP-1 cells and PBDM macrophages (**b**). Granularity of THP-1, THP-1PMA cells, and PBDM (**c**) macrophages. The ordinate axis represents the average value of the intensity of lateral light scattering by the cell in relative units (SSC). Relative mitochondrial content in THP-1, THP-1PMA cells, and PBDM macrophages (**d**). The mean fluorescence intensity of cells (arb. units) infused with MitoTracker Green FM is referred to as the MFI. The data are shown as the mean ± SD (*n* ≥ 3). * *p* < 0.05 in comparison to THP-1 cells.

Furthermore, microscopic examination revealed that more than 90% of THP-1PMA cells had a pronounced “fusiform” shape similar to PBDM. When studying the phenotype, it was shown that THP-1PMA cells reduce the number of cells carrying CD64 and increase the number of cells carrying CD11c, CD14, Cd36, and others, which is also characteristic of PBDM macrophages (Figure 4).

### 3.4. DCPD, OCP, and HAp Reduce the Phagocytic Activity of THP-1ATRA and THP-1PMA Cells

**Figure 5:**
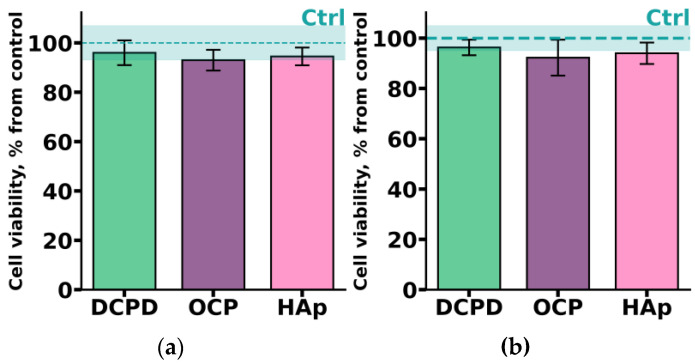
Viability of monocyte-like cells THP-1ATRA (**a**) and macrophage-like cells THP-1PMA (**b**) after 72 h of co-incubation with 1 mg/mL DCPD, OCP, or HAp. Ctrl—control (untreated cells). The data are presented as mean ± SD.

THP-1ATRA monocyte-like cells and THP-1PMA macrophage-like cells were incubated with 1 mg/mL DCPD, OCP, or HAp for 72 h. As shown in Figure 5, after incubation with the investigated CPs, the number of viable THP-1ATRA and THP-1PMA cells was more than 90%. Interestingly, we previously found that 1 mg/mL OCP, obtained in such a way, led to a significant decrease in the viability of C3H/10T1/2 mesenchymal cells [19].

Further in the study, we assessed the impact of DCPD, OCP, and HAp on key pro-inflammatory functions of human monocyte-like and macrophage-like cells, including phagocytic activity, acidic compartment content, as well as the production of ROS, NO, and pro-inflammatory cytokines.

Considering the crucial role of the phagocytic activity of monocytes and macrophages in the process of material biointegration and biodegradation, we initially investigated the influence of the examined calcium phosphate-based materials on the phagocytic activity of THP-1ATRA and THP-1PMA cells.

**Figure 6:**
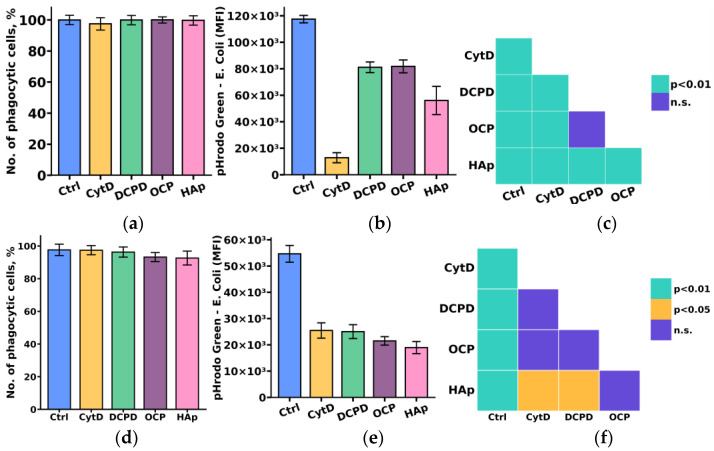
Number of phagocytic THP-1ATRA (**a**) and THP-1PMA cells (**b**); mean fluorescence intensity (MFI) of pHrodo Green *E. coli* in phagocytic THP-1ATRA (**d**) and THP-1PMA cells (**e**). Ctrl—control (untreated cells), CytD—cytochalasin D. The data are presented as mean ± SD. Statistical significance of differences in the fluorescence intensity of pHrodo Green *E. coli* in phagocytic THP-1ATRA (**c**) and THP-1PMA cells (**f**) among the investigated groups was determined using one-way ANOVA followed by Tukey’s test; n.s.—no significance.

The study results indicated that DCPD, OCP, and HAp reduced the phagocytic activity towards *pHrodo Green E. coli* in both monocyte-like THP-1ATRA and macrophage-like THP-1PMA cells, as evidenced by a decrease in fluorescent intensity of pHrodo Green *E. coli* in phagocytic cells. We found that the number of phagocytic cells of THP-1ATRA and THP-1PMA cells remained unchanged after 72 h of co-incubation with 1 mg/mL DCPD, OCP, or HAp compared to control cells that were not incubated with calcium phosphate-based materials, and it exceeded 95% (Figure 6a,d). In contrast, after 72 h of co-incubation with 1 mg/mL DCPD, OCP, or HAp, both THP-1ATRA and THP-1PMA cells exhibited a decrease in the MIF of pHrodo Green *E. coli* in phagocytic cells compared to control cells not incubated with calcium phosphate-based materials (Figure 6b,e). The effect was more pronounced in THP-1PMA cells. It is worth noting that HAp caused the most significant reduction in the MIF of pHrodo Green *E. coli* in phagocytic cells among the investigated cell variants.

Since the breakdown of large phagocytized particles, including bacteria, cellular debris, and dying cells, occurs in compartments such as lysosomes, we additionally investigated the impact of the examined calcium phosphate-based materials on the acidic compartment (lysosome) content in THP-1ATRA and THP-1PMA cells [40].

**Figure 7:**
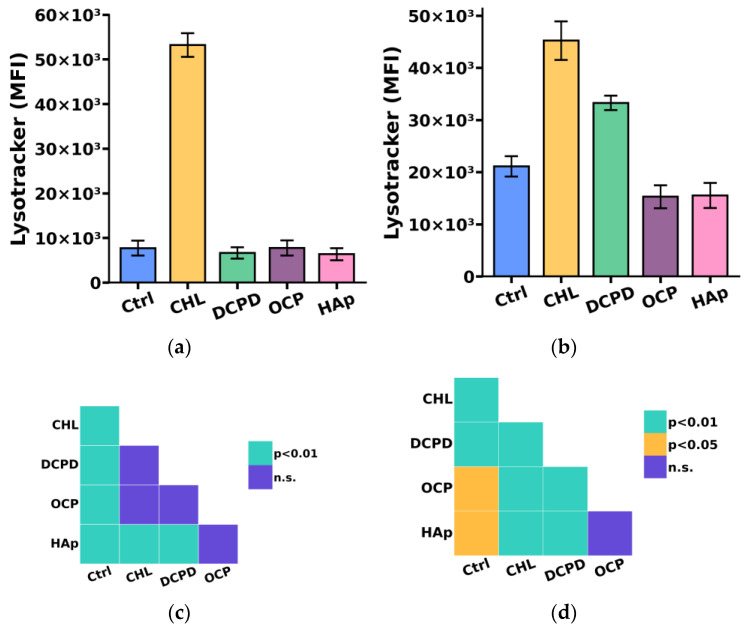
The acidic compartments (lysosomes) content in THP-1ATRA cells (**a**) and THP-1PMA cells (**b**) after 72 h of co-incubation with 1 mg/mL DCPD, OCP, or HAp. MFI—mean fluorescence intensity (a.u.). Ctrl—control (untreated cells); CHL—chloroquine. The data are presented as mean ± SD. (**c**,**d**) Statistical significance of differences in the content of acidic compartments in THP-1ATRA cells (**c**) and THP-1PMA cells (**d**) among the investigated groups, determined using one-way ANOVA followed by Tukey’s test. n.s.—no significance.

We found that the acidic compartment (lysosome) content in THP-1PMA cells slightly decreased after 72 h of co-incubation with 1 mg/mL OCP or HAp and increased after incubation with 1 mg/mL DCPD (Figure 7b,d) compared to control cells not incubated with calcium phosphate-based materials. In turn, the content of acidic compartments in THP-1ATRA cells remained unchanged compared to control cells after incubation with the examined calcium phosphate-based materials (Figure 7a,c).

### 3.5. DCPD, OCP, and HAp Affect the Intracellular Oxidative Activity of THP-1ATRA and THP-1PMA Cells

It is known that phagocytes produce reactive nitrogen species (NO) and reactive oxygen species (ROS), which enhance and regulate the immune response [41,42]. Reactive oxygen and nitrogen species are utilized by phagocytes to eliminate invading microorganisms, which can also cause damage to cells and tissues [41]. However, an excess of ROS and NO has been shown to lead to damage to normal body cells [41]. Therefore, in this study, we further investigated the impact of DCPD, OCP, and HAp on the oxidative intracellular activity of THP-1ATRA and THP-1PMA cells.

**Figure 8:**
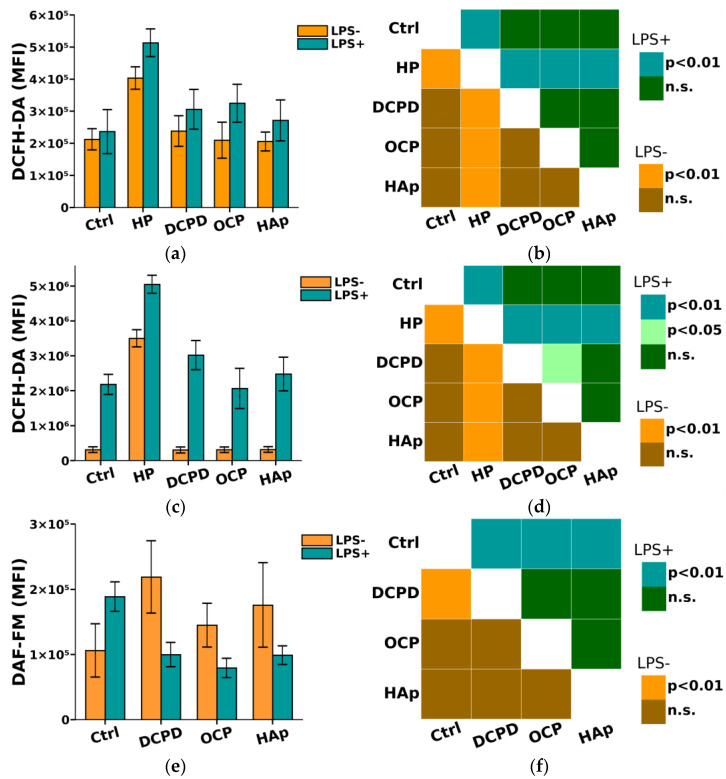

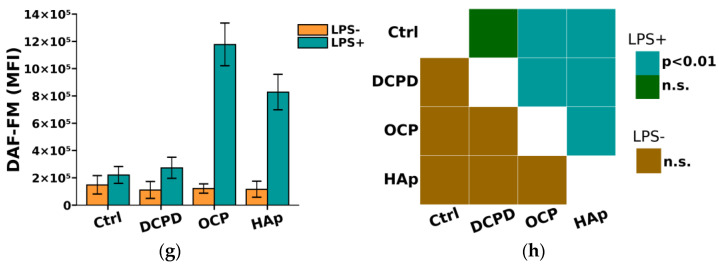
(**a**,**c**,**e**,**g**) Constitutive and LPS-induced intracellular ROS production by THP-1ATRA cells (**a**) and THP-1PMA cells (**b**) after 72 h of co-incubation with CPs; constitutive and LPS-induced intracellular NO production by THP-1ATRA cells (**e**) and THP-1PMA cells (**g**) after 72 h of co-incubation with CPs. MFI—mean fluorescence intensity of cell fluorescence (a.u.). Ctrl—control (untreated cells). HP—hydrogen peroxide. The data are presented as mean ± SD. (**b**,**d**,**f**,**h**) Statistical significance of differences in ROS production by THP-1ATRA cells (**b**) and THP-1PMA cells (**f**) among the investigated groups, determined using one-way ANOVA followed by Tukey’s test. n.s.—no significance. Statistical significance of differences in NO production by THP-1ATRA cells (**d**) and THP-1PMA cells (**h**) among the investigated groups, determined using one-way ANOVA followed by Tukey’s test. n.s.—no significance.

From Figure 8, it can be seen that DCPD, OCP, or HAp (1 mg/mL, 72 h) did not affect the constitutive intracellular production of ROS and NO by THP-1ATRA and THP-1PMA cells. Additionally, we found that DCPD, OCP, or HAp (1 mg/mL, 72 h) had no impact on LPS-induced intracellular ROS production by THP-1ATRA and THP-1PMA cells (Figure 8a–d). At the same time, LPS-induced intracellular NO production decreased after 72 h of co-incubation with 1 mg/mL DCPD, OCP, or HAp in THP-1ATRA cells (Figure 8e,f), remained unchanged after incubation with DCPD (1 mg/mL, 72 h), and increased after incubation with OCP or HAp (1 mg/mL, 72 h) in THP-1PMA cells (Figure 8g,h) compared to control cells not incubated with CPs.

### 3.6. DCPD, OCP, and HAp Affect the Secretion of Pro-Inflammatory Cytokines TNF-α, IL-1β, IL-6 by THP-1ATRA and THP-1PMA Cells 

Another important function of macrophages and monocytes is their ability to secrete numerous cytokines that play a crucial role in forming a pro-regenerative niche necessary for bone tissue restoration/regeneration [25]. As a result, the study analyzed the secretion of pro-inflammatory cytokines TNF-α, IL-1β, and IL-6 by THP-1ATRA and THP-1PMA cells after their incubation with the investigated calcium phosphate-based materials.

**Figure 9:**
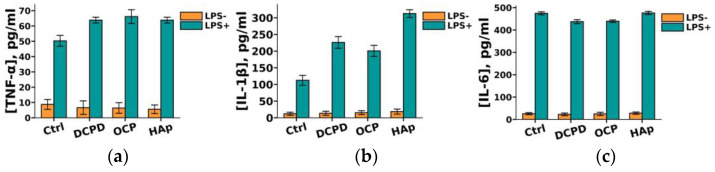

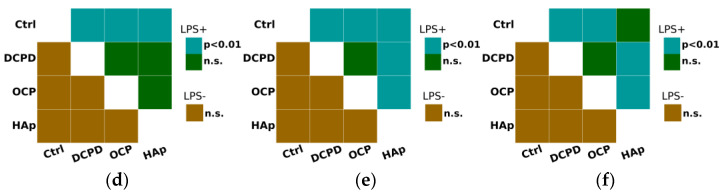
Constitutive and LPS-induced secretion of pro-inflammatory cytokines TNF-α (**a**), IL-1β (**b**), and IL-6 (**c**) by THP-1ATRA cells after 72 h of co-incubation with 1 mg/mL DCPD, OCP, or HAp. Ctrl—control (untreated cells). Concentration is presented per 5 × 10^4^ cells/mL. The data are presented as mean ± SD. (**d**–**f**) Statistical significance of differences in the production of pro-inflammatory cytokines TNF-α (**d**), IL-1β (**e**), and IL-6 (**f**) by THP-1ATRA cells among the investigated groups, determined using one-way ANOVA followed by Tukey’s test. n.s.—no significance.

It was demonstrated that DCPD, OCP, or HAp (1 mg/mL, 72 h) did not influence the constitutive secretion of pro-inflammatory cytokines TNF-α, IL-1β, and IL-6 but enhanced the LPS-induced secretion of TNF-α and IL-1β by THP-1ATRA cells compared to the control cells not incubated with the materials (Figure 9a,b). After incubation with DCPD, OCP, and HAp, the concentration of TNF-α in the culture medium of LPS-treated THP-1ATRA cells increased from 50 ± 4 pg/mL to 64 ± 2 pg/mL, 66 ± 5 pg/mL, and 64 ± 2 pg/mL, respectively. Following incubation with DCPD, OCP, and HAp, the concentration of IL-1β in the culture medium of LPS-treated THP-1ATRA cells increased from 113 ± 3 pg/mL to 226 ± 18 pg/mL, 201 ± 17 pg/mL, and 313 ± 9 pg/mL, respectively. Additionally, it was found that DCPD and OCP, but not HAp, slightly decreased LPS-induced IL-6 secretion by THP-1ATRA cells compared to control cells not incubated with the materials (Figure 9c). The concentration of IL-6 in the culture medium of LPS-treated THP-1ATRA cells after incubation with DCPD and OCP decreased from 475 ± 3 pg/mL to 437 ± 9 pg/mL and 439 ± 6 pg/mL, respectively.

**Figure 10:**
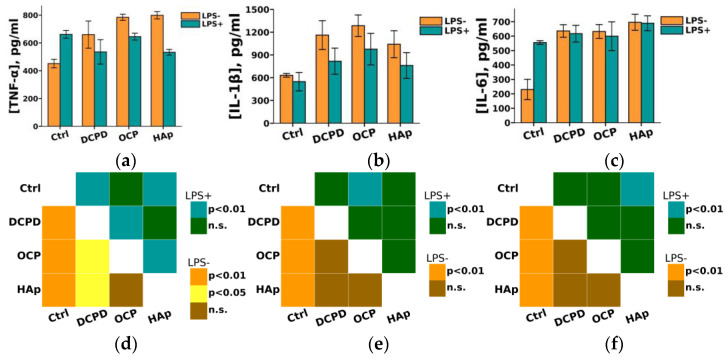
Constitutive and LPS-induced secretion of pro-inflammatory cytokines TNF-α (**a**), IL-1β (**b**), and IL-6 (**c**) by THP-1PMA cells after 72 h of co-incubation with 1 mg/mL DCPD, OCP, or HAp. Ctrl—control (untreated cells). Concentration is presented per 5 × 10^4^ cells/mL. The data are presented as mean ± SD. (**d**–**f**) Statistical significance of differences in the production of pro-inflammatory cytokines TNF-α (**d**), IL-1β (**e**), and IL-6 (**f**) by THP-1PMA cells among the investigated groups, determined using one-way ANOVA followed by Tukey’s test. n.s.—no significance.

Furthermore, we found that DCPD, OCP, or HAp (1 mg/mL, 72 h) did not affect LPS-induced secretion of pro-inflammatory cytokines IL-1β and IL-6 but increased their constitutive secretion by THP-1PMA cells compared to control cells not incubated with the materials (Figure 10). After incubation with DCPD, OCP, and HAp, the concentration of IL-6 in the culture medium of THP-1PMA cells increased from 230 ± 71 pg/mL (control values) to 636 ± 44 pg/mL, 632 ± 48 pg/mL, and 691 ± 113 pg/mL, respectively. Following incubation with DCPD, OCP, and HAp, the concentration of IL-1β in the culture medium of THP-1PMA cells increased from 631 ± 24 pg/mL (control values) to 1161 ± 190 pg/mL, 1285 ± 141 pg/mL, and 1041 ± 178 pg/mL, respectively. It was also observed that OCP and HAp, but not DCPD, increased the constitutive secretion of TNF-α and decreased its LPS-induced secretion by THP-1PMA cells compared to control cells not incubated with the materials (Figure 10a). The concentration of TNF-α in the culture medium of THP-1PMA cells after incubation with OCP and HAp increased from 451 ± 31 pg/mL to 786 ± 5 pg/mL and 799 ± 7 pg/mL, respectively, and decreased to 644 ± 18 pg/mL and 530 ± 6 pg/mL, respectively, in the culture medium of LPS-treated THP-1PMA cells.

## 4. Discussion

The development of biomaterials designed for the restoration of functions and regeneration of damaged bone tissue is currently one of the most important and relevant challenges in modern materials science. The specific structure and properties of various histotypes of bone tissue, as well as the processes of reparative histogenesis, impose high requirements on both materials and the technologies for their production. Ceramic calcium phosphate materials obtained under high-temperature synthesis conditions are known to be safe, non-toxic, and even biostable. However, they lack biointegration potential, not only failing to initiate regenerative processes but also causing fibrous encapsulation of the material, leading to a complete halt in the reparative osteogenesis process at the implantation site. Additionally, these materials exhibit comparative brittleness compared to native bone tissue, making them unsuitable for applications like dental implants or endoprostheses.

In contrast, the use of low-temperature calcium phosphate precursors, such as hydroxyapatite (HAp) and its precursors (amorphous phosphates, DCPD, TCP, OCP, etc.), synthesized under conditions reflecting natural (physiological) biomineralization processes, is a promising and rapidly developing approach. It is postulated that, like native calcium phosphates, these low-temperature calcium phosphates may have a direct inducing effect. These low-temperature CPs, including OCP (octacalcium phosphate) and DCPD (dicalcium phosphate dihydrate) are suggested to possess higher bioresorption rates and pronounced osteoinductive potentials compared to known analogs. The possibility of incorporating biological agents and performing targeted chemical functionalization of CPs directly during their synthesis, with the subsequent controlled release of necessary elements for the activation and maintenance of regenerative processes in the body, adds to the interest in these materials.

Despite the scientific interest and numerous studies in this field, knowledge and understanding of the impact of low-temperature ceramics on not only reparative histogenesis processes but also on immune activation and initiation of local aseptic inflammation, leading to rejection of CPs and osteolysis in the recipient bed, remain limited and insufficient.

Macrophages and monocytes are among the first cells to invade the surface of biomaterials after implantation and play an important role in their biodegradation via phagocytosis. [43,44]. Phagocytosis is one of the fundamental functions of monocytes and macrophages, and cellular-mediated resorption of calcium phosphate-based materials represents the central pathway for their in vivo degradation [45,46]. Monocyte-macrophage cells phagocytose damaged and apoptotic cells formed during the material implantation process and combat postoperative infection, thereby providing a favorable immune microenvironment for subsequent osteogenesis [25,47]. Our data on the influence of the investigated CPs on the phagocytic activity of monocyte-like and macrophage-like cells showed that the phagocytic index, that is, the number of cells capable of phagocytosis, did not change after incubation with the investigated CPs. However, all the studied CPs reduced the actual phagocytic activity, that is, the number of engulfed particles, in both cell types. Moreover, HAp caused the greatest reduction in phagocytic activity in both monocyte- and macrophage-like cells.

Macrophages are capable of phagocytosing relatively small particles (diameter less than 10 µm) of calcium phosphate [44]. It can be hypothesized that the reduction in phagocytic activity after incubation with the CPs investigated in this study may be associated with the uptake of these particles by cells and their subsequent biodegradation. Phagocytosis, like lysosomal biogenesis, is a Ca^2+^-dependent process [48,49]. An increase in lysosomal mass has been shown to be regulated by the activity of lysosomal channel TRPML1 [48]. When macrophages attach to particles, the TRPML1 channel opens in lysosomes, allowing Ca^2+^ to be released from lysosomes into the cytosol, causing lysosomal exocytosis at the phagocytic cup. This, in turn, increases the surface area of phagocytic macrophages and facilitates big particle engulfment [49,50]. In addition to lysosomal Ca^2+^, macrophages can also utilize an influx of extracellular Ca^2+^ released by dissolution on the “material-cell” surface during phagocytosis [51]. Notably, both extracellular and intracellular resorption pathways are characteristic of CP materials. The phagocytosis of small CP-particles with subsequent degradation inside phagolysosomes may disrupt the normal functioning of lysosomes by releasing excess Ca^2+^ and Pi, and by increasing osmotic pressure [52]. The possible consequence of these processes may be a disruption of normal lysosomal degradation, which, as already known, is crucial for sustainable phagocytosis in macrophages [53]. This mechanism potentially explains the decrease in lysosomal mass along with the inhibition of phagocytosis observed for the investigated OCP and HAp. Moreover, it is possible that DCPD, by comparison with OCP and HAp, can provide a more prolonged maintenance of lysosomal function and potentially has a greater ability for macrophage resorption. However, the described effects and their relationship with the physicochemical characteristics of specific CPs require further investigation.

Macrophages represent a heterogeneous cell population with a dynamic spectrum of functional states ranging from pro-inflammatory M1 to anti-inflammatory/immune-regulatory alternatively activated M2 macrophages, demonstrating pronounced differences in effector functions [54]. After the implantation of a biomaterial, during the acute inflammatory phase, macrophages initially polarize towards the M1 phenotype, secreting a series of characteristic pro-inflammatory factors such as TNFα, IL1β, IL6, reactive oxygen species (ROS), and nitric oxide (NO) [53,55]. However, the effective and timely transition from the M1 to the anti-inflammatory M2 phenotype defines bone tissue regeneration and implant integration [20]. During the phenotype switch from M1 to M2, anti-inflammatory cytokines such as BMP-2 (bone morphogenetic protein 2), vascular endothelial growth factor (VEGF), and IL-10 are released, creating the necessary osteogenic microenvironment for bone tissue regeneration [55]. Our results indicate that the increased induced, but not constitutive, intracellular production of NO after incubation with the investigated OCP and HAp suggests that these CPs may enhance the pro-inflammatory properties of macrophages, which could contribute to reducing the pathogenic load after material implantation. Elevated extracellular NO production may lead to contact damage to normal cells and intensify inflammatory processes; however, within the peri-implant site, NO production may contribute to angiogenesis, which is necessary for normal functioning of the implant and successful regenerative processes [42,56,57,58]. 

Another important function of macrophages and monocytes is their ability to secrete numerous cytokines that play a crucial role in the formation of a pro-regenerative niche necessary for bone tissue restoration/regeneration [25]. Bone healing begins with an inflammatory reaction that initiates the regeneration process, leading to the restoration of bone tissue under normal conditions [59]. However, an imbalanced immune reaction at this stage can disrupt the reparative process, ranging from delayed healing to the formation of non-regenerating bone defects [24]. During the inflammatory stage of bone tissue healing, the central cytokines secreted by macrophages and monocytes are IL-1, IL-6, and TNF-α [25]. The function of these pro-inflammatory cytokines is to create an osteogenic niche by recruiting and differentiating mesenchymal precursor cells into osteogenic cells [25,60,61,62].

The obtained data indicate that the investigated CPs do not influence the production of pro-inflammatory cytokines in non-activated monocyte-like cells. However, adding CPs to cells pre-activated by the Toll-like receptor ligand bacterial LPS significantly enhances the production of pro-inflammatory cytokines such as TNF and IL1-β. This enhancement of cytokine production may be related to the synergistic action of bacterial LPS and Ca^2+^ ions formed from CPs. The elevated levels of extracellular calcium are known to be detected near dying or activated cells, in areas of chronic infections, and are also formed during the dissolution and recrystallization of CPs [63,64]. The excess Ca^2+^ ions formed can serve as a danger signal and induce the assembly of the NLRP3 inflammasome in monocytes/macrophages through the activation of the calcium-sensing receptor (CaSR). In turn, this signaling cascade can be triggered by signals from Toll-like receptors [65,66]. NLRP3 inflammasome activation led to the production of IL-1β, with the highest production observed in cells incubated with HAp [67]. It is known that the activation of the NLRP-3 inflammasome and the production of IL1-β play important roles in the pathophysiology of hydroxyapatite-associated arthropathy [68]. Based on this information, it can be hypothesized that the pro-inflammatory properties of hydroxyapatite are primarily realized through the NLRP3 inflammasome activation and subsequent production of IL-1β [68]. Our findings align well with the clinically recognized issue of implant-associated infections, often leading to the rejection of bone implants [69]. Regarding CP materials, this phenomenon may be associated with the release of a large quantity of Ca^2+^ ions into the surrounding space during the interaction of cells with CPs, which, in conjunction with infectious agents, enhances the pro-inflammatory activation of monocytes.

Based on the obtained data, it can be seen that all investigated CPs are capable of increasing the production of pro-inflammatory cytokines in non-activated LPS macrophage-like cells. From the literature, it is known that calcium phosphate particles can act as DAMPs, contributing to the polarization of macrophages towards the M1 phenotype [70,71]. However, as previously stated, during material integration, macrophages initially polarize to the M1 phenotype, which exhibits pro-inflammatory behaviors by secreting typical pro-inflammatory cytokines, and only after that does the switch to the regenerative M2 phenotype occur [55,72]. Moreover, based on our data, it can be observed that with additional activation of these cells by LPS, there is no further increase in the production of the investigated cytokines upon the addition of CPs. In fact, the concentration of TNF-α and IL-1β even decreases. 

## 5. Conclusions

The necessity of developing bioactive and harmless materials imposes a challenge on modern scientists for a comprehensive express assessment of the bio- and immunocompatibility of new ceramic materials under in vitro conditions. Our obtained data suggest that the THP-1ATRA and THP-1PMA cell models can effectively and informatively reflect the monocytic macrophage response to the implanted calcium phosphate ceramics in in vitro conditions, demonstrating not only the harmlessness but also the immunomodulatory activity of the investigated materials. We believe that even such a simple model as the use of THP-1ATRA and THP-1PMA cells will allow scientists to promptly make the necessary adjustments to the composition of calcium phosphate materials directly in the development process ex tempore, as well as significantly save resources and reduce the use of laboratory animals.

The results presented in the study indicate that physiological participants in the process of bone tissue biomineralization—DCPD, OCP, and HAp, obtained by us using a low-temperature chemical transformation approach—may possess biological activity toward human macrophage and monocytic cells in vitro and potentially provide conditions necessary for bone tissue restoration/regeneration in the peri-implant site in vivo. Moreover, the most preferable calcium phosphate ceramic for implantation in patients with latent inflammation or unpredictable immune status appears to be DCPD, as this ceramic, in total, had the most favorable impact on the investigated cell models. To test this hypothesis, we will conduct further research on the influence of experimental low-temperature calcium phosphate ceramics on the immune status of laboratory SPF animals with modeled latent inflammation. 

## Data Availability

Data are available upon request from the authors.

## References

[1] Einhorn T.A., Gerstenfeld L.C. (2015). Fracture healing: Mechanisms and interventions. Nat. Rev. Rheumatol..

[2] Oryan A., Alidadi S., Moshiri A., Maffulli N. (2014). Bone regenerative medicine: Classic options, novel strategies, and future directions. J. Orthop. Surg. Res..

[3] Baldwin P., Li D.J., Auston D.A., Mir H.S., Yoon R.S., Koval K.J. (2019). Autograft, Allograft, and Bone Graft Substitutes: Clinical Evidence and Indications for Use in the Setting of Orthopaedic Trauma Surgery. J. Orthop. Trauma.

[4] Sohn H.-S., Oh J.-K. (2019). Review of bone graft and bone substitutes with an emphasis on fracture surgeries. Biomater. Res..

[5] Dorozhkin S.V. (2015). Calcium Orthophosphate-Containing Biocomposites and Hybrid Biomaterials for Biomedical Applications. J. Funct. Biomater..

[6] Kim S.-M., Kang I.-G., Cheon K.-H., Jang T.-S., Kim H.-E., Jung H.-D., Kang M.-H. (2020). Enhanced Bioactivity of Micropatterned Hydroxyapatite Embedded Poly(L-lactic) Acid for a Load-Bearing Implant. Polymers.

[7] Sahmani S., Khandan A., Esmaeili S., Saber-Samandari S., Ghadiri Nejad M., Aghdam M.M. (2020). Calcium phosphate-PLA scaffolds fabricated by fused deposition modeling technique for bone tissue applications: Fabrication, characterization and simulation. Ceram. Int..

[8] Levingstone T.J., Herbaj S., Dunne N.J. (2019). Calcium Phosphate Nanoparticles for Therapeutic Applications in Bone Regeneration. Nanomaterials.

[9] Rolvien T., Barbeck M., Wenisch S., Amling M., Krause M. (2018). Cellular Mechanisms Responsible for Success and Failure of Bone Substitute Materials. Int. J. Mol. Sci..

[10] Remes A., Williams D. (1992). Immune response in biocompatibility. Biomaterials.

[11] Valtanen R.S., Yang Y.P., Gurtner G.C., Maloney W.J., Lowenberg D.W. (2021). Synthetic and Bone tissue engineering graft substitutes: What is the future?. Injury.

[12] Wang W., Yeung K.W.K. (2017). Bone grafts and biomaterials substitutes for bone defect repair: A review. Bioact. Mater..

[13] Pankratov A.S., Fadeeva I.S., Minaychev V.V., O Kirsanova P., Senotov A.S., Yurasova Y.B., Akatov V.S. (2018). A biointegration of microand nanocrystalline hydroxyapatite: Problems and perspectives. Genes Cells.

[14] Szwed-Georgiou A., Płociński P., Kupikowska-Stobba B., Urbaniak M.M., Rusek-Wala P., Szustakiewicz K., Piszko P., Krupa A., Biernat M., Gazińska M. (2023). Bioactive Materials for Bone Regeneration: Biomolecules and Delivery Systems. ACS Biomater. Sci. Eng..

[15] Turnbull G., Clarke J., Picard F., Riches P., Jia L., Han F., Li B., Shu W. (2017). 3D bioactive composite scaffolds for bone tissue engineering. Bioact. Mater..

[16] Jitaru S., Hodisan I., Timis L., Lucian A., Bud M. (2016). The use of bioceramics in endodontics—Literature review. Med. Pharm. Rep..

[17] Wei S., Ma J.-X., Xu L., Gu X.-S., Ma X.-L. (2020). Biodegradable materials for bone defect repair. Mil. Med. Res..

[18] Hench L.L., Polak J.M. (2002). Third-generation biomedical materials. Science.

[19] Teterina A.Y., Smirnov I.V., Fadeeva I.S., Fadeev R.S., Smirnova P.V., Minaychev V.V., Kobyakova M.I., Fedotov A.Y., Barinov S.M., Komlev V.S. (2021). Octacalcium Phosphate for Bone Tissue Engineering: Synthesis, Modification, and In Vitro Biocompatibility Assessment. Int. J. Mol. Sci..

[20] Fadeeva I.S., Teterina A.Y., Minaychev V.V., Senotov A.S., Smirnov I.V., Fadeev R.S., Smirnova P.V., Menukhov V.O., Lomovskaya Y.V., Akatov V.S. (2023). Biomimetic Remineralized Three-Dimensional Collagen Bone Matrices with an Enhanced Osteostimulating Effect. Biomimetics.

[21] Teterina A.Y., Minaychev V.V., Smirnova P.V., Kobiakova M.I., Smirnov I.V., Fadeev R.S., Egorov A.A., Ashmarin A.A., Pyatina K.V., Senotov A.S. (2023). Injectable Hydrated Calcium Phosphate Bone-like Paste: Synthesis, In Vitro, and In Vivo Biocompatibility Assessment. Technologies.

[22] Zhao T., Chu Z., Ma J., Ouyang L. (2022). Immunomodulation Effect of Biomaterials on Bone Formation. J. Funct. Biomater..

[23] Okamoto K., Takayanagi H. (2019). Osteoimmunology. Cold Spring Harb. Perspect. Med..

[24] Loi F., Córdova L.A., Pajarinen J., Lin T., Yao Z., Goodman S.B. (2016). Inflammation, fracture and bone repair. Bone.

[25] Niu Y., Wang Z., Shi Y., Dong L., Wang C. (2020). Modulating macrophage activities to promote endogenous bone regeneration: Biological mechanisms and engineering approaches. Bioact. Mater..

[26] Longoni A., Knežević L., Schepers K., Weinans H., Rosenberg A.J.W.P., Gawlitta D. (2018). The impact of immune response on endochondral bone regeneration. NPJ Regen. Med..

[27] Cai B., Lin D., Li Y., Wang L., Xie J., Dai T., Liu F., Tang M., Tian L., Yuan Y. (2021). N2-Polarized Neutrophils Guide Bone Mesenchymal Stem Cell Recruitment and Initiate Bone Regeneration: A Missing Piece of the Bone Regeneration Puzzle. Adv. Sci..

[28] Joshi A., Soni A., Acharya S. (2022). In vitro models and ex vivo systems used in inflammatory bowel disease. Vitr. Model..

[29] Baxter E.W., Graham A.E., Re N.A., Carr I.M., Robinson J.I., Mackie S.L., Morgan A.W. (2020). Standardized protocols for differentiation of THP-1 cells to macrophages with distinct M(IFNγ+LPS), M(IL-4) and M(IL-10) phenotypes. J. Immunol. Methods.

[30] Cong L., Gao Z., Zheng Y., Ye T., Wang Z., Wang P., Li M., Dong B., Yang W., Li Q. (2020). Electrical stimulation inhibits Val-boroPro-induced pyroptosis in THP-1 macrophages via sirtuin3 activation to promote autophagy and inhibit ROS generation. Aging (Albany N. Y.).

[31] Chanput W., Mes J.J., Wichers H.J. (2014). THP-1 cell line: An in vitro cell model for immune modulation approach. Int. Immunopharmacol..

[32] Lomovskaya Y.V., Kobyakova M.I., Senotov A.S., Lomovsky A.I., Minaychev V.V., Fadeeva I.S., Shtatnova D.Y., Krasnov K.S., Zvyagina A.I., Akatov V.S. (2022). Macrophage-like THP-1 cells derived from high-density cell culture are resistant to trail-induced cell death via down-regulation of death-receptors DR4 and DR5. Biomolecules.

[33] Lomovskaya Y.V., Kobyakova M.I., Senotov A.S., Fadeeva I.S., Lomovsky A.I., Krasnov K.S., Shtatnova D.Y., Akatov V.S., Fadeev R.S. (2023). Myeloid differentiation increases resistance of leukemic cells to trail-induced death by reducing the expression of DR4 and DR5 receptors. Biochem. Moscow Suppl. Ser. A.

[34] Kobyakova M., Lomovskaya Y., Senotov A., Lomovsky A., Minaychev V., Fadeeva I., Shtatnova D., Krasnov K., Zvyagina A., Odinokova I. (2022). The Increase in the Drug Resistance of Acute Myeloid Leukemia THP-1 Cells in High-Density Cell Culture Is Associated with Inflammatory-like Activation and Anti-Apoptotic Bcl-2 Proteins. Int. J. Mol. Sci..

[35] Terpilowski M.A. (2019). Scikit-posthocs: Pairwise multiple comparison tests in Python. J. Open Source Softw..

[36] Berzina-Cimdina L., Borodajenko N. (2012). Research of calcium phosphates using Fourier transform infrared spectroscopy. Infrared Spectrosc.-Mater. Sci. Eng. Technol..

[37] Pourpoint F., Gervais C., Bonhomme-Coury L., Azaïs T., Coelho C., Mauri F., Alonso B., Babonneau F., Bonhomme C. (2007). Calcium Phosphates and Hydroxyapatite: Solid-State NMR Experiments and First-Principles Calculations. Appl. Magn. Reson..

[38] Tseng Y.-H., Mou C.-Y., Chan J.C.C. (2006). Solid-State NMR Study of the Transformation of Octacalcium Phosphate to Hydroxyapatite: A Mechanistic Model for Central Dark Line Formation. J. Am. Chem. Soc..

[39] Davies E., Duer M.J., Ashbrook S.E., Griffin J.M. (2012). Applications of NMR Crystallography to Problems in Biomineralization: Refinement of the Crystal Structure and ^31^P Solid-State NMR Spectral Assignment of Octacalcium Phosphate. J. Am. Chem. Soc..

[40] Lee H.-J., Woo Y., Hahn T.-W., Jung Y.M., Jung Y.-J. (2020). Formation and Maturation of the Phagosome: A Key Mechanism in Innate Immunity against Intracellular Bacterial Infection. Microorganisms.

[41] Hoebe K., Janssen E., Beutler B. (2004). The interface between innate and adaptive immunity. Nat. Immunol..

[42] Santos S.S., Brunialti M.K.C., Rigato O., Machado F.R., Silva E., Salomao R. (2012). Generation of nitric oxide and reactive oxygen species by neutrophils and monocytes from septic patients and association with outcomes. Shock.

[43] Ogle M.E., Segar C.E., Sridhar S., Botchwey E.A. (2016). Monocytes and Macrophages in Tissue Repair: Implications for Immunoregenerative Biomaterial Design. Exp. Biol. Med..

[44] Sheikh Z., Abdallah M.-N., Hanafi A.A., Misbahuddin S., Rashid H., Glogauer M. (2015). Mechanisms of *In Vivo* Degradation and Resorption of Calcium Phosphate Based Biomaterials. Materials.

[45] Lu J., Descamps M., Dejou J., Koubi G., Hardouin P., Lemaitre J., Proust J.-P. (2002). The Biodegradation Mechanism of Calcium Phosphate Biomaterials in Bone. J. Biomed. Mater. Res..

[46] Kloc M., Kubiak J.Z. (2021). The Role of Monocytes and Macrophages in Homeostasis and Disease and Novel Avenues for Putative Treatments. Int. J. Mol. Sci..

[47] Kyriakides T.R., Foster M.J., Keeney G.E., Tsai A., Giachelli C.M., Clark-Lewis I., Rollins B.J., Bornstein P. (2004). The CC chemokine ligand, CCL2/MCP1, participates in macrophage fusion and foreign body giant cell formation. Am. J. Pathol..

[48] Cao Q., Yang Y., Zhong X.Z., Dong X.-P. (2017). The lysosomal Ca2+ release channel TRPML1 regulates lysosome size by activating calmodulin. J. Biol. Chem..

[49] Nunes P., Demaurex N. (2010). The role of calcium signaling in phagocytosis. J. Leukoc. Biol..

[50] Tang T., Yang Z.-Y., Wang D., Yang X.-Y., Wang J., Li L., Wen Q., Gao L., Bian X.-W., Yu S.-C. (2020). The role of lysosomes in cancer development and progression. Cell Biosci..

[51] Liu X., Wang N., Zhu Y., Yang Y., Chen X., Chen Q., Zhou H., Zheng J. (2016). Extracellular Calcium Influx Promotes Antibacterial Autophagy in Escherichia Coli Infected Murine Macrophages via CaMKKβ Dependent Activation of ERK1/2, AMPK and FoxO1. Biochem. Biophys. Res. Commun..

[52] Liu Z., Xiao Y., Chen W., Wang Y., Wang B., Wang G., Xu X., Tang R. (2014). Calcium Phosphate Nanoparticles Primarily Induce Cell Necrosis through Lysosomal Rupture: The Origination of Material Cytotoxicity. J. Mater. Chem. B.

[53] Wong C.-O., Gregory S., Hu H., Chao Y., Sepúlveda V.E., He Y., Li-Kroeger D., Goldman W.E., Bellen H.J., Venkatachalam K. (2017). Lysosomal Degradation Is Required for Sustained Phagocytosis of Bacteria by Macrophages. Cell Host Microbe.

[54] Agoro R., Taleb M., Quesniaux V.F.J., Mura C. (2018). Cell iron status influences macrophage polarization. PLoS ONE.

[55] Xiao L., Shiwaku Y., Hamai R., Tsuchiya K., Sasaki K., Suzuki O. (2021). Macrophage Polarization Related to Crystal Phases of Calcium Phosphate Biomaterials. Int. J. Mol. Sci..

[56] Das A., Abas M., Biswas N., Banerjee P., Ghosh N., Rawat A., Khanna S., Roy S., Sen C.K. (2019). A Modified Collagen Dressing Induces Transition of Inflammatory to Reparative Phenotype of Wound Macrophages. Sci. Rep..

[57] Rahat M.A., Hemmerlein B. (2013). Macrophage-tumor cell interactions regulate the function of nitric oxide. Front. Physiol..

[58] Ziche M., Morbidelli L. (2000). Nitric Oxide and Angiogenesis. J. Neuro-Oncology.

[59] Schmidt-Bleek K., Schell H., Schulz N., Hoff P., Perka C., Buttgereit F., Volk H.-D., Lienau J., Duda G.N. (2012). Inflammatory phase of bone healing initiates the regenerative healing cascade. Cell Tissue Res..

[60] Glass G.E., Chan J.K., Freidin A., Feldmann M., Horwood N.J., Nanchahal J. (2011). TNF-α promotes fracture repair by augmenting the recruitment and differentiation of muscle-derived stromal cells. Proc. Natl. Acad. Sci. USA.

[61] Yang X., Ricciardi B.F., Hernandez-Soria A., Shi Y., Camacho N.P., Bostrom M.P. (2007). Callus mineralization and maturation are delayed during fracture healing in in terleukin-6 knockout mice. Bone.

[62] Ma T., Miyanishi K., Trindade M.C.D., Genovese M., Regula D., Smith R.L., Goodman S.B. (2003). Interleukin 1 receptor antagonist inhibits localized bone formation in vivo. J. Rheumatol..

[63] Murthy S., Karkossa I., Schmidt C., Hoffmann A., Hagemann T., Rothe K., Seifert O., Anderegg U., von Bergen M., Schubert K. (2022). Danger Signal Extracellular Calcium Initiates Differentiation of Monocytes into SPP1/Osteopontin-Producing Macrophages. Cell Death Dis..

[64] Tang Z., Li X., Tan Y., Fan H., Zhang X. (2018). The Material and Biological Characteristics of Osteoinductive Calcium Phosphate Ceramics. Regen. Biomater..

[65] Rossol M., Pierer M., Raulien N., Quandt D., Meusch U., Rothe K., Schubert K., Schöneberg T., Schaefer M., Krügel U. (2012). Extracellular Ca2+ Is a Danger Signal Activating the NLRP3 Inflammasome through G Protein-Coupled Calcium Sensing Receptors. Nat. Commun..

[66] Lee G.-S., Subramanian N., Kim A.I., Aksentijevich I., Goldbach-Mansky R., Sacks D.B., Germain R.N., Kastner D.L., Chae J.J. (2012). The Calcium-Sensing Receptor Regulates the NLRP3 Inflammasome through Ca^2+^ and cAMP. Nature.

[67] Wang Y., Liu X., Shi H., Yu Y., Yu Y., Li M., Chen R. (2020). NLRP3 inflammasome, an immune-inflammatory target in pathogenesis and treatment of cardiovascular diseases. Clin. Transl. Med..

[68] Jin C., Frayssinet P., Pelker R., Cwirka D., Hu B., Vignery A., Eisenbarth S.C., Flavell R.A. (2011). NLRP3 inflammasome plays a critical role in the pathogenesis of hydroxyapatite-associated arthropathy. Proc. Natl. Acad. Sci. USA.

[69] Dong J., Wang W., Zhou W., Zhang S., Li M., Li N., Pan G., Zhang X., Bai J., Zhu C. (2022). Immunomodulatory Biomaterials for Implant-Associated Infections: From Conventional to Advanced Therapeutic Strategies. Biomater. Res..

[70] Velard F., Braux J., Amedee J., Laquerriere P. (2013). Inflammatory Cell Response to Calcium Phosphate Biomaterial Particles: An Overview. Acta Biomater..

[71] Mahon O.R., Kelly D.J., McCarthy G.M., Dunne A. (2020). Osteoarthritis-Associated Basic Calcium Phosphate Crystals Alter Immune Cell Metabolism and Promote M1 Macrophage Polarization. Osteoarthr. Cartil..

[72] Brown B.N., Badylak S.F. (2013). Expanded Applications, Shifting Paradigms and an Improved Understanding of Host—Biomaterial Interactions. Acta Biomater..

